# A New Wearable System for Postural Balance Assessment: Comparison with EquiTest and Static Posturography in Healthy Adults

**DOI:** 10.3390/audiolres16020045

**Published:** 2026-03-17

**Authors:** Valerio Maria Di Pasquale Fiasca, Alfredo Gabriele Nanni, Marco Pozzi, Lorenzo Collino, Barbara Martino, Paolo Ranieri, Eliana Filipponi, Giulio Dehesh, Andrea Beghi, Federica Di Berardino

**Affiliations:** 1 Department of Clinical Sciences and Community Health, University of Milan, Via della Commenda 19, 20122 Milan, Italy; 2 Audiology Unit, Department of Surgical Sciences, Foundation IRCCS Ca’ Granda Ospedale Maggiore Policlinico, 20122 Milan, Italy; 3 Department of Neurology, A. Perrino’s Hospital, 72100 Brindisi, Italy; 4 Otolaryngology Unit, Aerospace Medicine Institute “A. Mosso”, Italian Air Force, 20122 Milan, Italy; 5 Direzione Aziendale Professioni Sanitarie, Fondazione IRCCS Ca’ Granda Ospedale Maggiore Policlinico, 20122 Milan, Italy; 6 Independent Researcher, Via Chiesanuova 239, 35136 Padova, Italy; 7 Neurotology and Vestibular Rehabilitation Clinic—Centro di Medicina, 44124 Ferrara, Italy

**Keywords:** Svep, EquiTest Gravity, SOT, IMU-based posturography, stability, posturography, balance assessment, inertial wearable sensors, computerised dynamic posturography

## Abstract

**Background**: Objective assessment of postural control is central to the clinical evaluation of vestibular disorders. Although force-platform-based posturography is considered the gold standard, its use may be limited by cost and infrastructural requirements. Wearable inertial measurement units (IMUs) represent a promising alternative; however, their clinical validation should account for intrinsic differences in measurement paradigms rather than strict metric equivalence. **Objective**: To preliminarily evaluate the within-session reliability of a wearable IMU-based medical device for balance assessment (Gravity), and its agreement with established static (SBP) and computerised dynamic posturographic systems (CDP) in healthy subjects. **Methods**: Sixty-three healthy adults were enrolled in two independent method comparison studies: a wearable IMU-based balance system versus a static stabilometric platform (GRAVITY vs. SVEP; *n* = 42) and a wearable IMU-based balance system versus computerised dynamic posturography (Gravity vs. EquiTest; *n* = 21). Gravity measurements were obtained simultaneously with reference systems across standardised sensory conditions. Within-session reliability and method agreement were assessed. **Results**: Within-session reliability of Gravity was outcome-dependent. Length-based components demonstrated higher repeatability (ICC (single) = 0.25–0.35; ICC (average) = 0.41–0.52), with narrower limits of agreement (LoA = ±9–12%) and lower measurement error (SEM = 3.3–4.3%). In comparison with SBP, length-based measures exhibited narrower limits (LoA = ±12–17) and more consistent relationships. Comparison with CDP revealed moderate agreement for composite and preferential scores (LoA: −2.20–7.07; −5.54–8.12). **Conclusions**: Gravity sensor may represent a clinically meaningful, outcome-dependent performance, with superior reliability and comparability for length-based postural measures compared with area-based measures. The device could provide balance assessments compatible with both static and dynamic posturographic systems, accounting for physiological variability. These findings support the potential clinical use of wearable IMU-based posturography, particularly in settings where conventional force-platform systems are not readily available, and warrant further validation in larger, more clinically diverse populations.

## 1. Introduction

Postural control assessment plays a central role in the clinical evaluation of vestibular and neurological disorders [1,2,3,4,5]. Techniques such as computerised dynamic posturography and foam tests provide critical data on centre-of-pressure (COP) [6,7] and postural sway. Established approaches include computerised dynamic posturography and static stabilometry, which quantify balance using force-platform-based measurements and controlled sensory perturbations. Although these systems are widely validated, their use is limited by high costs, dedicated infrastructure requirements, and reduced accessibility in routine clinical settings. Since 2002, wearable inertial measurement units (IMUs) have emerged as promising low-cost alternatives for objective human movement assessment [8,9,10] and represent an emerging alternative, enabling postural assessment through trunk kinematics under ecological conditions [11]. However, IMU-based systems rely on fundamentally different measurement models compared to force platforms [12]. Consequently, their validation should not aim at strict metric equivalence, but rather at demonstrating compatibility within physiological variability and controlled measurement dispersion [13]. The present study aimed to evaluate whether Gravity, a wearable IMU-based medical device, provides balance measurements compatible with both dynamic and static posturographic reference systems, as commonly used in clinical practice (the gold standard), while explicitly accounting for intrinsic differences in technology and measurement paradigms in healthy subjects.

## 2. Materials and Methods

In the present study, a total of 63 healthy subjects volunteered to participate: 21 healthy adult subjects were included in the CDP–Gravity and 42 in the Gravity–SBP comparisons. The two study samples were independent and recruited for different method comparison analyses. They were engaged in regular physical activity and had no self-reported orthopaedic or neurological problems that negatively affected their balance and/or mobility. All participants fell within CDP normative ranges and reported no history of balance disorders or vertigo. Patients with untreated visual disorders were ruled out. Study group characteristics are reported in detail in Section 3.

### 2.1. Gravity System

**Figure 1:**
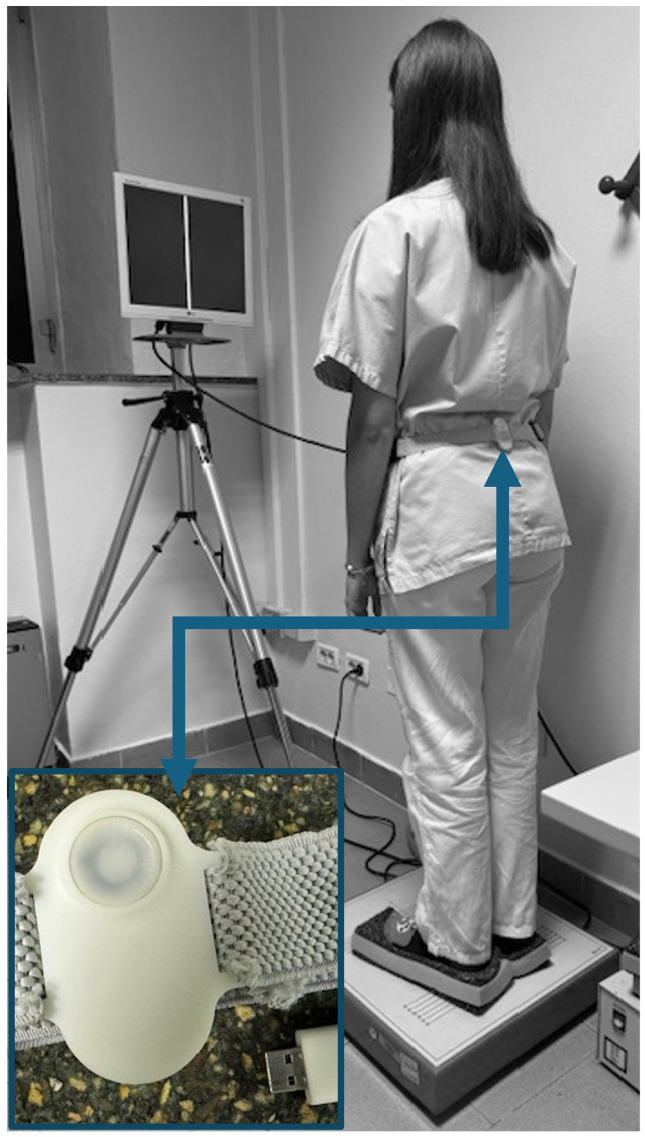
Positioning of Gravity sensor device.

Gravity is a wearable medical device consisting of a triaxial accelerometer, gyroscope, and magnetometer, integrated into a single IMU (Cilia Biotech Srl, Alessandria, Italy). The sensor is factory-calibrated to the manufacturer’s specifications. For posturographic static assessment, Gravity was configured with a sampling frequency of 100 Hz and a low-pass filter of 50 Hz. Raw sensor data are processed in real time by a sensor fusion algorithm implemented in the proprietary Venice software (Release 1.0.0.9), supplied with the device. This processing pipeline is designed to reduce measurement noise and allow a stable projection of the centre-of-gravity (COG) trajectory. Gravity is worn at the level of L5, a position selected to minimise bias due to respiratory movements, reduce artefacts related to accidental limb or upper-body motion, and approximate whole-body centre-of-mass (COM) dynamics. The device communicates via Bluetooth Low Energy (BLE) and is designed to ensure a guaranteed battery life of approximately 32 h of continuous use. Figure 1 shows the device’s position.

### 2.2. CDP: EquiTest

The NeuroCom SMART EquiTest Clinical Research System (Natus Medical international, Clackamas, OR, USA) is the gold standard procedure to investigate the relative contributions of vestibular, visual and somatosensory inputs [14]. The SMART EquiTest includes an 18″ dual force plate and a visual surround, in which withers are fixed or moveable (rotating up/down or forward) in reference to the participant’s sway with the Data Acquisition Toolkit software (version 9.2). To investigate the relative contributions of vestibular, visual, and somatosensory inputs, the analysis was based on the Clinical Test of Sensory Interaction of Balance, or ‘‘Foam and Dome’’, as suggested by Cook and Horak [15] and first proposed by Nashner et al. in 1982 [16]. For the EquiTest–Gravity comparison, only the visual (deceptive visual surround) and proprioceptive (sway-referenced dynamic platform) perturbation systems of EquiTest were used for both the simultaneous measurements. This choice was made to ensure that both systems were evaluated under equivalent sensory perturbation conditions, assuming comparable external challenges. Therefore, four Sensory Organisation Test (SOT) conditions are included: conditions 1 (eyes opened, EO) and 2 (eyes closed, EC) with a fixed platform, and conditions 4 (EO) and 5 (EC) with a sway-referenced platform.

### 2.3. SBP: SVEP

SVEP (Politecnica, Modena, Italy) is a static stabilometric force platform that measures centre-of-pressure displacement during quiet stance. Gravity–SVEP comparisons were conducted to evaluate compatibility between trunk-based kinematic measures and force-platform-based postural sway measures. This is a stable force plate, mounted on 3 strain-gauge force transducers positioned at the vertices of an equilateral triangle, which is sensitive to vertical force and provides a description of body sway in terms of the patient’s centre-of-pressure displacement. Tests were carried out using a fixed visual target placed at a distance of 1.20 m, at eye level. X, Y and the absolute centre-of-pressure displacements from the projection of a theoretical barycentre and the sway area (SX), sway length (LX), and sway velocity (VM) were recorded at 5 Hz, over 51.2 s, using the Svep software (Svep 6.4). The subjects underwent static posturography in each of four conditions—stable condition with visual fixation (eyes opened, EO), without visual fixation (eyes closed, EC), unstable condition on foam pads (Orsafoam, Gorla Minore, Italy) with visual fixation (PAD EO) and without visual fixation (PAD EC)—in order to assess the relative contributions of vestibular, visual and somatosensory inputs, as reported by Di Berardino et al. [17]. Platform-based systems estimate COM sway area by projecting point-based COP measurements onto the ground. The Gravity sensor, positioned at the L5 level and therefore close to the COM, enables more direct and accurate estimation of sway area and exhibits lower variability in sway length. Sway length is derived through a sensor fusion algorithm based on an inverted pendulum model to project movement onto the ground plane. Despite these methodological differences, length-based outcomes were comparable across systems.

### 2.4. Study Protocol

At the beginning of the evaluation, each participant underwent a medical examination performed by a specialised neurotologist, including medical history, otoscopy, and a bedside balance examination (including detection of spontaneous or positional nystagmus, cerebellar function testing, vestibulo-spinal and vestibulo-ocular reflexes testing, as well as analysis of vestibular functional symmetry). Before the test began, a detailed explanation of the procedure was provided. Participants were required to remove their shoes, though they could keep their socks if preferred. With the EquiTest, once positioned on the platform, each participant was secured with a safety harness to minimise the risk of falls during the test.

Gravity measurements were performed simultaneously during SVEP and EquiTest analysis. Throughout the test, participants were instructed to look straight ahead, remain calm, keep their arms relaxed at their sides, and maintain their feet firmly on the platform. Each condition was repeated twice, with approximately one minute of rest between trials.

The study was conducted in accordance with the Strengthening the Reporting of Observational Studies in Epidemiology (STROBE) Statement, guidelines for reporting observational studies, and with the Helsinki Declaration. The study was performed under the Milan University Research Ethics Board guidelines for retrospective observational study on healthy subjects. No formal research ethics board approval was necessary, and therefore, no reference number was generated. The participation in the study was voluntary, and the subjects were not paid for it. Informed consent was obtained from all subjects involved in the study. The subjects’ anonymity has been guaranteed.

### 2.5. Statistical Analysis

All statistical analyses were performed using R (R Foundation for Statistical Computing, Vienna, Austria) [18]. Demographic and anthropometric variables were summarised using descriptive statistics. Continuous variables are reported as mean ± standard deviation when normally distributed and as median with interquartile range when non-normally distributed. Categorical variables are reported as counts and percentages. For both the comparisons, outcome values were calculated as the average of Trial 1 and Trial 2 for each participant to provide a more stable estimate and reduce the risk of bias. To evaluate the stability of the Gravity test, Trial 1 and Trial 2 were compared within the same session in participants with complete data. Scatterplots and Spearman correlation test were applied to preliminarily analyse the relationship between variables. Within-session reliability was separately quantified to characterise measurement error. An intraclass correlation coefficient (ICC) based on a two-way mixed-effects model with absolute agreement was applied. Single-measure ICC [ICC(3,1)] and average-measure ICC [ICC(3,2)] were calculated for Trial 1 and Trial 2, reflecting the reliability of one measurement and of repeated measurements, respectively. In addition, Bland–Altman analysis was used to evaluate the agreement between Trial 1 and Trial 2 of the Gravity test, providing information on systematic biases and on the expected range of differences between repeated measurements for an individual subject. Measurement error was further quantified using the standard error of measurement (SEM), quantifying the typical measurement error in percentage points. The minimal detectable change at the 95% confidence level (MDC95) was calculated to identify the smallest change that can be interpreted as a real change beyond measurement error. To compare the new Gravity test with the established SBP (SVEP) test, analyses were performed using the average of Trial 1 and Trial 2 for each method. Agreement between both Gravity vs. SVEP and Gravity vs. EquiTest was primarily assessed using Bland–Altman analysis. Overall agreement between the two tests was further summarised using Lin’s concordance correlation coefficient (CCC), which reflects how closely the measurements from Gravity and SVEP or EquiTest match across participants. To examine whether differences between the tests changed across the range of measurements, an explorative Deming regression was performed.

## 3. Results

A total of 63 patients were included in the study; 42 subjects were included in the comparison between Gravity and sbp (SVEP), whereas 21 were included for the analysis of Gravity and EquiTest, with no overlap between the two groups. Demographic and anthropometric characteristics are reported in Table 1. The subjects were mostly females. Age and most anthropometric variables showed non-normal distributions and are therefore reported as median and interquartile range. Conversely, the height showed a normal distribution and is reported as mean and standard deviation.

### 3.1. Descriptive Analysis and Comparison of Gravity with SBP (SVEP)

Descriptive statistics for Gravity and SVEP outcomes are reported in Table 2. The results for each patient were calculated as the mean of Trial 1 and Trial 2 to reduce the risk of random error in the comparison between the two tests. The resulting three sensory components (visual, vestibular and somatosensory) are expressed as percentages of a total (sum = 100%), implying interdependence among components. For the Gravity test, area-based components showed greater variability (8–10%) compared with length-based components (3–4%). A similar pattern was observed for SVEP, with area components displaying wider dispersion than length components. Overall, length-derived measures demonstrated more homogeneous distributions across participants for both tests. The exploratory Spearman correlation analysis with 95% bootstrap CI revealed weak-to-moderate associations between Gravity and SVEP, indicating that participants with higher SVEP values generally exhibited higher Gravity values, albeit with inter-individual variability.

**Figure 2:**
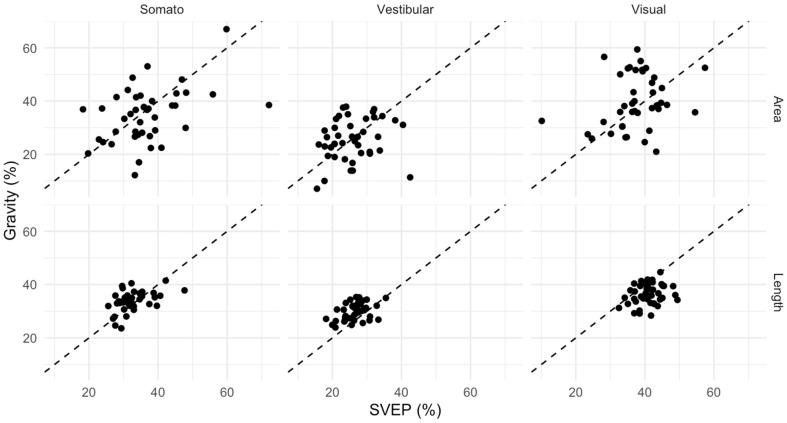
Scatterplots analysing the relationship between Gravity and SVEP. Dashed lines represent equal percentage in both SVEP and Gravity testing.

To provide a clinical description of the relationship between Gravity and SVEP, supportive analyses were performed using the mean of Trial 1 and Trial 2 for each method. Visual inspection of scatterplots showed a positive association between Gravity and SVEP across measures and sensory components, although with substantial dispersion around the line of identity, particularly for area-based outcomes. Dispersion was visibly reduced for length-based measures. These results are also reported in Figure 2.

### 3.2. Within Session—Reliability of Gravity

Within-session reliability of the Gravity test was assessed by comparing Trial 1 and Trial 2 in 41 participants with complete data who underwent SVEP and Gravity testing (Table 3). For area-based components, reliability was poor, with ICC (single) values ranging from 0.16 to 0.21 and wide 95% confidence intervals, frequently crossing zero. Bland–Altman analysis showed minimal systematic bias across components; however, limits of agreement were wide (approximately ±25–35%), indicating substantial within-subject variability. Measurement error was high, with SEM values between 9.0% and 12.2% and MDC95 values exceeding 25%. In contrast, length-based components demonstrated higher repeatability. ICC (single) values ranged from 0.25 to 0.35, while ICC (average) values increased to 0.41–0.52, indicating improved reliability when averaging the two trials. Bland–Altman bias remained close to zero for all components, with substantially narrower limits of agreement (approximately ±9–12%). Correspondingly, SEM values ranged from 3.3% to 4.3%, and MDC95 values ranged from 9.3% to 11.9%.

### 3.3. Method Comparison Between Gravity and SVEP

Method comparison analyses between Gravity and SVEP, based on the mean of Trial 1 and Trial 2, are presented in Table 4. Bland–Altman analysis revealed small mean biases for area-based components (ranging from −1.94% to 2.25%), indicating little systematic over- or underestimation by Gravity compared with SVEP. However, limits of agreement were wide (approximately ±18–24 percentage points), suggesting limited agreement at the individual level. Lin’s concordance correlation coefficients were low to moderate (CCC = 0.20–0.42), indicating poor overall concordance. Deming regression analyses showed evidence of proportional bias for vestibular and visual AREA components, with slopes substantially greater than one and large negative intercepts, indicating increasing divergence between methods at higher values. For length-based components, Bland–Altman bias was small (−4.61% to 3.44%), and limits of agreement were notably narrower than for area-based measures (approximately ±7–14 percentage points). Concordance was modest, with CCC values ranging from 0.14 to 0.43. Deming regression revealed limited proportional bias for length-based components, with slopes closer to unity and smaller intercepts compared with area-based measures, suggesting a more consistent relationship between Gravity and SVEP.

### 3.4. Comparison of Gravity with EquiTest

**Figure 3:**
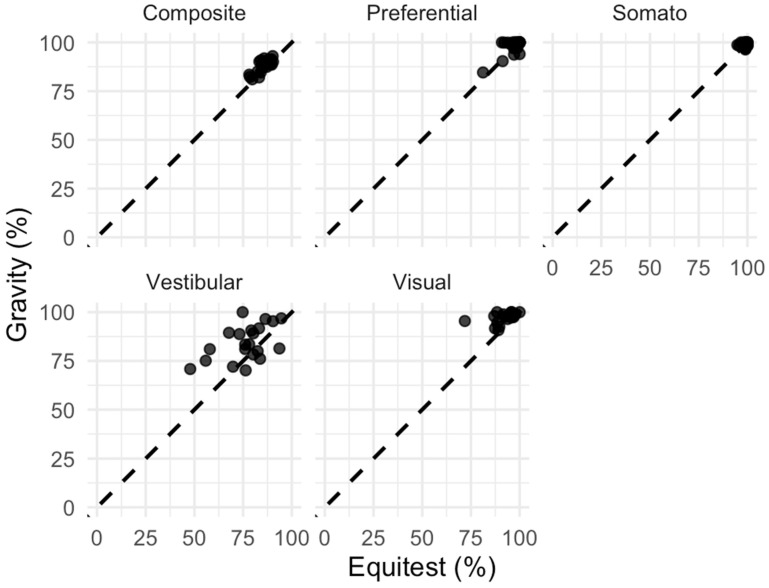
Scatterplots analysing the relationship between Gravity and EquiTest. Dashed lines represent equal percentage in both SVEP and Gravity testing.

Descriptive statistics for Gravity and EquiTest SOT outcomes are reported in Table 5. All variables are expressed as percentages and are presented as median and interquartile range due to non-normal distributions and pronounced ceiling effects. Composite and preferential scores showed high median values in both methods, approaching the upper limit of the scale. In contrast, vestibular and visual components exhibited a wider distribution, indicating greater inter-individual variability. Overall, Gravity yielded slightly higher median values than EquiTest across all components, with the largest absolute differences observed for the vestibular and visual components. Scatterplots revealed a generally positive association between Gravity and EquiTest across all SOT components, although with variable dispersion and ceiling effect (as depicted in Figure 3). Spearman correlation analysis showed a strong association for the composite score (ρ = 0.72, 95% CI 0.38–0.89), and moderate associations for preferential (ρ = 0.52, 95% CI 0.09–0.82), visual (ρ = 0.49, 95% CI 0.06–0.79), and vestibular components (ρ = 0.43, 95% CI −0.04–0.77). No meaningful association was observed for the somatosensory component (ρ = −0.01, 95% CI −0.40–0.38). Bland–Altman between Gravity and EquiTest analyses are subsequently summarised. Mean between-method differences (Gravity–EquiTest) were small for the somatosensory (bias = 0.17 percentage points) and preferential components (bias = 1.29 percentage points), indicating minimal systematic bias at the group level. Composite scores showed a modest positive bias (2.43 percentage points), with relatively narrow limits of agreement. In contrast, larger biases were observed for the vestibular (7.81 percentage points) and visual components (4.84 percentage points), indicating a tendency for Gravity to yield higher values than EquiTest in these domains. Limits of agreement were notably wider for vestibular (−13.18 to 28.81 percentage points) and visual components (−5.49 to 15.16 percentage points), suggesting variability in individual-level agreement between methods. Concordance was moderate for the composite (CCC = 0.64) and preferential (CCC = 0.65) scores, indicating fair agreement between Gravity and EquiTest. Lower concordance was observed for vestibular (CCC = 0.38) and visual components (CCC = 0.24), while concordance for the somatosensory component was negligible (CCC = 0.02).

## 4. Discussion

The balance and the related adaptive responses to changes in environmental context are not based on a fixed set of equilibrium reflexes but on a flexible functional motor skill that can adapt with training and experience [19,20]. Several tests have been developed [21,22] to analyse this complex function and are commercially available for clinical and research purposes. Dynamic and static posturography are useful assessments for patients with various types of imbalances. It is well known that posturographic results exhibit intrinsic variability [13,17,23,24], which makes their interpretation for clinical use challenging [2]. The use of wearable devices has been previously reported as capable of reliably measuring balance and postural stability, although validation studies and comparisons with other kinds of devices are lacking [14,25]. This preliminary study investigated the properties of Gravity, a novel balance assessment system. We evaluated its within-session reliability and its agreement with two established reference methods (SVEP and EquiTest) in healthy individuals. We aimed to explore the characteristics that could guide the path to the optimal use of the Gravity device.

Within-session reliability of the Gravity system was outcome-dependent. Area-based measures showed low repeatability. Conversely, length-based measures demonstrated higher reliability, with improved ICC values, narrower limits of agreement, and lower SEM and MDC_95_. These findings support the preferential use of length-derived outcomes for clinical assessment and longitudinal monitoring. In the comparison with the STP (SVEP), Gravity showed weak-to-moderate associations with small mean biases but wide limits of agreement, particularly for area-based components. Length-based measures exhibited narrower limits of agreement and more consistent between-method relationships, indicating better comparability. Proportional bias was observed for some area-based vestibular and visual components. Comparison with CDP (EquiTest) showed moderate agreement for composite and preferential scores, supporting the possible use of Gravity as a global postural assessment tool. In contrast, agreement for vestibular and visual components was lower and characterised by wider limits of agreement, likely due to ceiling effects and reduced inter-individual variability in healthy subjects. These findings warrant further validation in clinical populations with greater balance impairment.

The Gravity–SVEP comparison used sensory input perturbation conditions closely aligned across all four conditions of the modified Clinical Test of Sensory Interaction on Balance (m-CTSIB). In contrast, the Gravity–EquiTest comparison relied exclusively on CDP’s perturbation system. This allowed simultaneous testing of the same participant with both systems under identical external perturbations, enabling a direct comparison of measurements obtained under equivalent sensory challenges.

Statistical analyses revealed the presence of bias in the visual and vestibular outcomes in the comparison between EquiTest and Gravity. Nevertheless, Gravity showed a reduction in outcome values under conditions 4 and 5, consistent with expected clinical behaviour. These parameters were characterised by low inter-subject variability. This finding suggests that Gravity and EquiTest, due to their inherently different measurement principles and sensing approaches, may be associated with systematic differences in the normative scaling of the recorded outcomes. This assumption should be further investigated in future studies by including datasets from patients with balance disorders. Furthermore, it should include the assessment of sensitivity and specificity of the respective outcomes, while explicitly accounting for the structural and methodological differences between the two systems.

For EquiTest measurements, the dataset—although limited in size—was acquired without prior familiarisation or preparatory trials. EquiTest normative data are usually based on three trials. The first trial, in the absence of prior familiarisation, exhibited marked differences compared with the second trial and was therefore deemed unusable. It is possible that the EquiTest data retained for analysis (second trial only) were still influenced by emotional or adaptation-related effects. Such effects may have introduced postural alterations not directly related to sensory input processing.

The present preliminary findings suggest that Gravity could provide stable and physiologically plausible balance measurements with similarities to both dynamic and static posturographic reference systems. From a clinical perspective, further studies are needed to confirm Gravity outcomes as reliable measurements within a single session. The use of IMU, such as Gravity, may enable an ecological, low-cost, and logistically affordable assessment of balance function [12,26,27,28,29]. Nonetheless, the force plate remains the gold standard for obtaining reliable balance measurements. This type of test could also represent a valuable option in low-resource facilities.

The use of IMU, often mounted on the lower trunk, was successful in identifying several gait and posture differences in previous reports [8,12,30]. Some studies have also proposed the use of IMU in place of platform-based tests [31], and tested their role in comparison with the latter in the diagnosis and rehabilitation of balance-impaired patients [32,33]. The inclusion of two independent reference systems (SVEP and EquiTest), analysed in separate samples, strengthens the external relevance of the findings. When evaluating the results of testing the Gravity sensor against both a sensor-based system and a widely used force-platform-based test, a conceptual distinction between the different techniques should be considered. Some of the observed discrepancies could be inherent to biomechanical and methodological differences rather than device inaccuracy. The Gravity sensor is indeed placed closer to the COM rather than to the COP, which is farther from the COG. This may yield greater measurement stability because of the shorter distance. In contrast, COP-based analyses could be more sensitive to foot length and ankle work. Conversely, it provides a broader perspective on its performance across different measurement principles. The next analyses require a comparison of test results in pathological subjects. Our preliminary analysis, however, suggests that the Gravity metric may be consistent with the measurements reported by the other systems.

In the present study, we separated reliability analyses from method comparison analyses. Trial 1 and Trial 2 were analysed explicitly to characterise the within-session repeatability of Gravity, whereas method comparison analyses used the average of repeated trials to reduce the influence of random measurement error. Some limitations should be acknowledged in this preliminary analysis. First, sample sizes were relatively small. Moreover, pronounced ceiling effects were observed for several SOT-derived components, particularly for composite, preferential, and somatosensory scores. Future investigations should include larger datasets, including a wide cohort of healthy and balance-impaired patients, and the use of three trials per condition to allow a more robust and clinically oriented validation of the Gravity–EquiTest comparison. Nevertheless, the present preliminary analysis remains informative as an initial assessment of the robustness and dispersion of the measurements obtained with the two systems.

## 5. Conclusions

The results of our preliminary report indicate that Gravity exhibits clinically outcome-dependent performance, with superior repeatability for length-based measures compared with area-based measures. The wearable IMU-based medical device could provide balance measurements compatible with both dynamic and static posturographic systems within an acceptable physiological range. These results attesting to the stability of portable IMU might be a hint to a future implementation in the diagnostic process and rehabilitative therapy of vestibular impairment. We support its use for postural assessment, particularly in settings where traditional posturography is not readily available. In addition, further evaluation of Gravity in larger and clinically diverse populations, and validation analyses, are warranted to determine its clinical applicability and to establish clinically meaningful thresholds for agreement.

## Figures and Tables

**Table 1:** Demographic and anthropometric characteristics of the samples.

Study Samples	Variable	Value
SVEP (*n* = 42)	Sex	F: 35 (83.3%); M: 7 (16.7%) *
	Age (years)	33.0 [27.0–50.5] ^+^
	Weight (kg)	60.0 [57.0–70.0] ^+^
	Height (cm)	166.8 ± 7.7 ^†^
	Sensor height (cm)	99.0 [96.0–102.0] ^+^
EquiTest (*n* = 21)	Sex	M: 13 (61.9%); F: 8 (38.1%) *
	Age (years)	26.0 [24.0–54.0] ^+^
	Weight (kg)	69.8 ± 9.5 ^†^
	Height (cm)	175.0 ± 11.1 ^†^

* *n* (%); ^+^ median [IQR]; ^†^ mean (SD).

**Table 2:** Descriptive statistics for Gravity and SVEP (mean of Trial 1 and Trial 2, *n* = 42).

Measure	Component	Test	Results	Spearman ρ (95% CI)
Area	Somatosensory	Gravity	34.71 ± 10.25 ^†^	0.387 [0.104–0.643]
		SVEP	35.15 [32.18–39.13] ^+^	
	Vestibular	Gravity	25.48 ± 7.84 ^†^	0.241 [−0.077–0.522]
		SVEP	25.79 ± 6.57 ^†^	
	Visual	Gravity	39.82 ± 10.10 ^†^	0.261 [−0.075–0.545]
		SVEP	37.55 [34.13–42.26] ^+^	
Length	Somatosensory	Gravity	33.66 ± 3.76 ^†^	0.424 [0.121–0.665]
		SVEP	31.59 [29.64–34.26] ^+^	
	Vestibular	Gravity	29.95 ± 3.19 ^†^	0.406 [0.087–0.664]
		SVEP	26.51 ± 3.59 ^†^	
	Visual	Gravity	36.39 ± 3.93 ^†^	0.211 [−0.083–0.478]
		SVEP	41.00 ± 3.81 ^†^	

^†^ mean ± SD; ^+^ median [IQR].

**Table 3:** Within-session reliability for Gravity (Trial 1 vs. Trial 2, *n* = 41).

Measure	Component	ICC Single (95% CI)	ICC Average (95% CI)	Bland–Altman Bias (LoA)	SEM	MDC95
Area	Somatosensory	0.177 [−0.14–0.459]	0.301 [−0.326–0.629]	1.16 (−32.1–34.42)	11.98	33.20
	Vestibular	0.206 [−0.109–0.482]	0.342 [−0.245–0.651]	1.03 (−24.01–26.08)	9.02	25.00
	Visual	0.162 [−0.152–0.445]	0.278 [−0.357–0.616]	−2.19 (−36.06–31.68)	12.21	33.85
Length	Somatosensory	0.354 [0.054–0.595]	0.523 [0.103–0.746]	−0.52 (−10.1–9.05)	3.45	9.55
	Vestibular	0.271 [−0.032–0.53]	0.426 [−0.065–0.692]	0.88 (−8.37–10.13)	3.34	9.26
	Visual	0.254 [−0.06–0.521]	0.405 [−0.127–0.685]	−0.36 (−12.26–11.55)	4.28	11.87

**Table 4:** Method comparison between Gravity and SVEP.

Measure	Component	Bland–Altman Bias (LoA)	Lin’s Coefficient (CCC)	Deming	
				Intercept	Slope
Area	Somatosensory	−1.94 (−23.5–19.62)	0.418	−1.607	0.991
	Vestibular	−0.32 (−18.22–17.59)	0.202	−31.106	2.194
	Visual	2.25 (−19.78–24.29)	0.229	−49.757	2.385
Length	Somatosensory	1.17 (−7.4–9.74)	0.434	12.010	0.666
	Vestibular	3.44 (−3.77–10.66)	0.270	10.017	0.752
	Visual	−4.61 (−13.93–4.71)	0.142	−9.926	1.130

**Table 5:** Descriptive statistics of comparison between Gravity and EquiTest subjects and association tests with Spearman correlation, Bland–Altman agreement and Lin’s concordance correlation coefficient (CCC).

Component	Method	Median [IQR]	Spearman ρ (95% CI)	Bias	LoA	CCC
Composite	EquiTest	85.00 [83.33–88.33]	0.720 [0.377–0.885]	2.43	−2.20–7.07	0.643
	Gravity	88.58 [85.08–90.82]				
Preferential	EquiTest	98.26 [95.30–100.00]	0.516 [0.091–0.824]	1.29	−5.54–8.12	0.653
	Gravity	100.00 [97.31–100.00]				
Somatosensory	EquiTest	98.95 [97.92–100.00]	−0.007 [−0.396–0.377]	0.17	−3.36–3.71	0.016
	Gravity	99.04 [98.34–100.00]				
Vestibular	EquiTest	78.12 [73.12–83.16]	0.427 [−0.037–0.771]	7.81	−13.18–28.81	0.377
	Gravity	83.39 [78.30–90.63]				
Visual	EquiTest	94.51 [89.13–95.83]	0.487 [0.062–0.786]	4.84	−5.49–15.16	0.241
	Gravity	97.97 [96.50–99.05]				

LoA: Limits of agreement.

## Data Availability

The original contributions presented in this study are included in the article. Further inquiries can be directed to the corresponding author.

## Abbreviations

The following abbreviations are used in this manuscript:

CDP	Computerised dynamic posturography
SBP	Static balance platform
IMU	Inertial measurement units
CoP	Centre-of-pressure
CoM	Centre-of-mass
EO	Eyes opened
EC	Eyes closed
